# Cellular Damage in the Target and Out-Of-Field Peripheral Organs during VMAT SBRT Prostate Radiotherapy: An In Vitro Phantom-Based Study

**DOI:** 10.3390/cancers14112712

**Published:** 2022-05-30

**Authors:** Igor Piotrowski, Katarzyna Kulcenty, Wiktoria Suchorska, Marcin Rucinski, Karol Jopek, Marta Kruszyna-Mochalska, Agnieszka Skrobala, Piotr Romanski, Adam Ryczkowski, Dorota Borowicz, Natalia Matuszak, Julian Malicki

**Affiliations:** 1 Department of Electroradiology, Poznan University of Medical Sciences, Ul. Garbary 15, 61-866 Poznan, Poland; wiktoria.suchorska@wco.pl (W.S.); marta.kruszyna-mochalska@wco.pl (M.K.-M.); agnieszka.skrobala@wco.pl (A.S.); adam.ryczkowski@wco.pl (A.R.); natalia.matuszak@interia.pl (N.M.); julian.malicki@wco.pl (J.M.); 2 Radiobiology Laboratory, Department of Medical Physics, Greater Poland Cancer Centre, Ul. Garbary 15, 61-866 Poznan, Poland; katarzyna.kulcenty@wco.pl; 3 Department of Histology and Embryology, Poznan University of Medical Sciences, 60-781 Poznan, Poland; marcinruc@ump.edu.pl (M.R.); kjopek@ump.edu.pl (K.J.); 4 Department of Medical Physics, Greater Poland Cancer Centre, Ul. Garbary 15, 61-866 Poznan, Poland; piotr.romanski@wco.pl (P.R.); dorota.borowicz@wco.pl (D.B.)

**Keywords:** prostate cancer, stereotactic body radiation therapy, volumetric arc therapy, out-of-field radiation, dosimetry, DNA damage, cell death, gene expression profile

## Abstract

**Simple Summary:**

New developments show that patients with prostate cancer can benefit from radiotherapy delivered with a hypo-fractionated regimen. The aim of our study was to investigate the effect of hypo-fractionated stereotactic body radiation therapy (SBRT) of prostate cancer on out-of-field organs. We used a humanoid phantom to irradiate prostate cells in conditions similar to patient therapy, using SBRT planning. Our results show that radiation doses in the location of the intestine and lung resulted in significantly higher radiation doses than the further locations. We observed a high radiotoxic effect in the cells irradiated in the prostate, and a small increase in DNA damage and cell killing in the intestine location. Gene expression analysis revealed significant enrichment of the biological processes related to the radiation response in the prostate. In the lung and thyroid, the enrichment of several gene groups was revealed, however the processes were not clearly related to the response to radiation. Our study provides extensive data on out-of-field safety of prostate SBRT.

**Abstract:**

Hypo-fractionated stereotactic body radiation therapy (SBRT) is an effective treatment for prostate cancer (PCa). Although many studies have investigated the effects of SBRT on the prostate and adjacent organs, little is known about the effects further out-of-field. The aim of this study was to investigate, both in vitro and in a quasi-humanoid phantom, the biological effects (using a dose-scaling approach) of radiation in the out-of-field peripheral organs delivered by 6 MV volumetric modulated arc therapy (VMAT) SBRT in a prostate cancer model. Healthy prostate cells were irradiated in a phantom at locations corresponding to the prostate, intestine, lung, thyroid, and brain. Seven 10 Gy fractions of VMAT SBRT were delivered to the target in a single session without intermission (scaled-up method). Radiochromic films were used to measure the doses. The radiobiological response was assessed by measuring DNA breaks, the cell survival fraction, and differences in gene expression profile. Our results showed a strong, multiparametric radiobiological response of the cells in the prostate. Outside of the radiation field, the highest doses were observed in the intestine and lung. A small increase (not statistically significant) in DNA damage and cell death was observed in the intestines. Several gene groups (cell cycle, DNA replication) were depleted in the lung and thyroid (DNA replication, endocytosis), but further analysis revealed no changes in the relevant biological processes. This study provides extensive evidence of the types and extent of radiobiological responses during VMAT SBRT in a prostate cancer model. Additional research is needed to determine whether the radiobiological effects observed in the peripheral organs are validated in a clinical context.

## 1. Introduction

Prostate cancer (PCa) is the third most common cancer worldwide, and the second most common cancer among men [1]. Treatment is commonly multimodal, including external beam radiation therapy (EBRT). Prostate cancer has a low α/β value and is thus amenable to hypo-fractionated radiotherapy techniques, such as stereotactic body radiation therapy (SBRT) [2]. The cumulative dose for SBRT typically ranges from 30 to 50 Gy, with dose per fractions ranging from 7 to 10 Gy [3,4]. These radiation schemes are often delivered with increasingly complex delivery techniques, such as volumetric modulated arc therapy (VMAT), which provide better dose conformity, and thus lower toxicity to adjacent organs [5]. Studies on SBRT up to 50 Gy show that this method can be applied with relative safety, providing that threshold dose constraints for the nearby organs are considered [6].

Although the use of intensity-modulated beams for SBRT yields satisfactory dose conformity, they also increase the doses to organs and tissues distant from the target (i.e., peripheral organs), due to the changing gantry locations during delivery, particularly when non-coplanar techniques are used. The radiation energy drops rapidly beyond the radiation field and, as Kirkby et al. have suggested, it is the low energy component that contributes to the higher radiobiological effectiveness [7,8]. In recent years, several studies have investigated the effect of VMAT prostate radiotherapy on the organs adjacent to the target, with clinical data showing good sparing of the rectum and bladder. In addition, in vitro studies have investigated the cellular response outside the primary beam, up to several cm from the field edge [5,9,10]. A few studies have measured the doses and radiation energy in peripheral organs located more distant from the target [11,12]. Those studies have shown that some peripheral organs may receive total doses of up to 1 Gy (depending on the distance from the target) during radiotherapy delivered with these advanced techniques. Dosimetric studies can measure the radiation doses and provide estimates of the risk of secondary cancers in peripheral organs (using statistical modelling); there is a notable lack of comprehensive data on the biological effects of out-of-field radiation in locations at a greater distance from the target. Moreover, it is difficult to reliably determine the potential biological effects of doses up to 1 Gy from scattered low energy radiation further away from the field during VMAT. Many variables—the size of the target volume, dose, and dose rate—influence organ response to radiation, and studies show that even doses as low as a few Gy can induce toxicity and increase the risk of radiation-induced secondary tumours over time [13,14].

In this context, the aim of this study was to investigate in vitro and in a phantom model of prostate cancer the DNA damage and differential mRNA expression from this damage response in out-of-field organs after 6 MV VMAT SBRT (dose-scaling approach). Although we expected the cells in peripheral organs to be exposed to low radiation doses, existing research suggests higher radiotoxicity of out-of-field radiation, which could induce various biological effects, depending on the location [7,8].

## 2. Materials and Methods

### 2.1. Cell Culture

The PNT1A cell line (normal human prostate epithelial cells immortalised with SV40) was obtained from the European Collection of Authenticated Cell Cultures. Cells were cultured at 37 °C in a humidified atmosphere with 5% carbon dioxide in air. The PNT1A cells were cultured in RPMI 1640 medium (Biowest, Nuaillé, France) with 10% foetal bovine serum (FBS; Biowest, Nuaillé, France) and 1% penicillin/streptomycin 10,000 U/mL (Merck Millipore, Darmstadt, Germany). Cells were grown in T-25 Nunc™ culture flasks without a filter (ThermoFisher Scientific, Waltham, MA, USA). For irradiation, the flasks were filled with phosphate buffer saline (PBS; Biowest, Nuaillé, France) directly before irradiation, to ensure homogenous dose distribution.

### 2.2. Radiation Delivery

The PNT1A cells were placed and irradiated in a quasi-humanoid phantom under conditions similar to those of real-life radiotherapy. The phantom was made with water-equivalent poly(methyl methacrylate) (PMMA) slices in the coronal plane to simulate the torso, head, and neck. The slices included natural cork and gypsum inhomogeneities in the lung and head locations to simulate lung tissue and bones, respectively. The layer for cell irradiation was equipped with a water container to allow for the placement of five flasks with the cells. The characteristics of this custom-built phantom were described in detail elsewhere [15].

**Figure 1:**
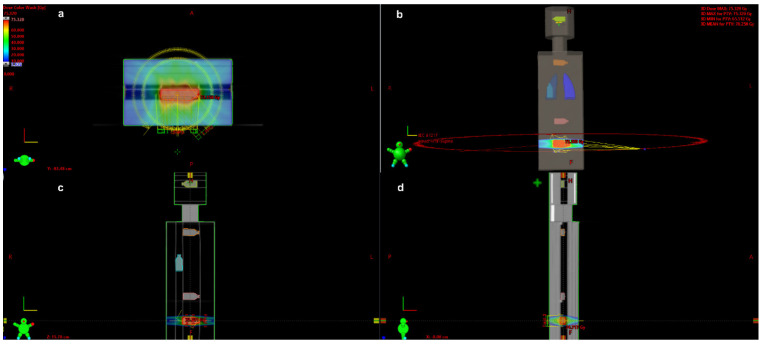
Transversal, 3D coronal, coronal and sagittal view of three co-planar arcs for VMAT plans. (**a**) transversal view; (**b**) 3D coronal view; (**c**) coronal view; (**d**) sagittal view. The plans were created to treat the PNT1A cells in the prostate location and to simulate the organs at risk for the following: intestine (red); right lung (blue); thyroid (orange); and brain (yellow).

A radiation treatment plan was created for the T-25 culture flask containing PNT1A cells in the prostate location, which was delineated as the gross tumour volume (GTV). The flask was considered the clinical target volume (CTV). To create the planning target volume (PTV), a margin of 3 mm was added around the CTV, following the usual clinical practice to ensure that the PTV received the prescribed dose. The other four flasks were placed in the water containers in the following locations at various distances from the axial plane: intestine (transverse colon) (15 cm from the axial plane; lung (35 cm); thyroid (52 cm); and brain (74 cm). Figure 1 shows the VMAT plan for these organs at risk.

We used a 6 MV photon beam with a flattening filter to deliver the total dose of 70 Gy to the cells in the prostate, delivered in seven consecutive fractions of 10 Gy each (the highest fraction dose delivered for prostate SBRT). This approach is known as dose scaling (see AAPM Report 158) [16]. The 10 Gy SBRT fraction was used for planning (instead of 2 Gy, which is used for conventional fractionations, to a total dose of 70 Gy) because we wanted to assure the same scheme of collimator leaves’ movement (a different scheme is used for different doses per fraction during arc therapies). Treatment plans were prepared for the RapidArc VMAT technique using three co-planar arcs, with the first, second, and third arcs delivered, respectively, counterclockwise (gantry angles from 140° to 220°), clockwise (181° to 179°), and counterclockwise (179° to 181°), and with a 0.5 cm multi-leaf collimator (Figure 1).

By scaling the dose to 70 Gy (in target) in a single delivery, we were able to obtain more reliable measurements of the out-of-field doses using Gafchromic EBT3 films (dynamic dose range: 0.01 Gy to 20 Gy, optimum 0.2 Gy to 10 Gy), especially for locations as far away as the thyroid and brain [16]. This approach also allowed us to compare the biological effects induced by this radiation delivery method, especially for the different out-of-field locations. The radiation doses to these “organs” (in the phantom) were due to scattered radiation, thus the term “nontarget dose” was equivalent to “out-of-field” dose.

A 6 MV beam was delivered with a TrueBeam linear accelerator (Varian Medical Systems, Palo Alto, CA, USA). Optimisation and calculations were performed in the Eclipse Treatment Planning System, v. 15.6 (Varian Medical Systems, Palo Alto, CA, USA), using the anisotropic analytical algorithm.

Before irradiation, dosimetry pre-verification was performed, using the electronic portal imaging device system and the gamma evaluation method (γ passing rate ≥ 95%, 3%/2 mm, a 10% threshold, global normalisation).

### 2.3. Cell Irradiation Setup

PNT1A cells were cultured and irradiated in T-25 Nunc™ culture flasks (ThermoFisher Scientific, Waltham, MA, USA). The flasks were filled with room temperature PBS (Biowest, Nuaillé, France) directly before irradiation to ensure homogenous dose distribution during delivery. Cells were irradiated in the quasi-humanoid phantom described above. The culture flasks were placed in the water container in the phantom at a depth of 11 cm in five locations (distance from axial plane in cm) to simulate the following organs: prostate (PTV, central beam axis (CAX)); intestine (15 cm); lung (35 cm); thyroid (52 cm ); and brain (74 cm). The cells in the prostate were irradiated to a total dose of 70 Gy (10 Gy delivered seven times, consecutively) in approximately 50 min. In parallel, sham-irradiated cells were cultured, filled with PBS, and kept at room temperature (they were not exposed to radiation). After irradiation, the cells were handled as described in Section 2.5, Section 2.6 and Section 2.7. The experiment was performed in at least three biological replicates.

### 2.4. Dose Measurements

Gafchromic films were used to measure the delivered dose to the organs at risk (OAR). GAFChromic™ EBT3 radiographic films were cut into pieces to fit the bottom of a T-25 cell culture flask (6 × 3.5 cm) and marked to ensure the same orientation throughout the experiment. Before irradiation, the film pieces were wrapped in plastic wrap to avoid contact with water and affixed to the bottom of the flasks to be irradiated and the unirradiated control flasks. The distance between the film and cell layer was approximately 1 mm. The EBT3 films were used to measure the out-of-field radiation doses, as these films are more sensitive than previous Gafchromic films, their response shows low dependence on energy and they also have a better optical density (dose range for EBT3 films: 0.01–10 Gy).

A rigorous protocol was developed and used to read and calibrate the dosimetric films, in accordance with the criteria of Report 235 of the American Association of Physicists in Medicine (AAPM) [17]. The EBT3 films were calibrated from 1 cGy to 200 cGy. The doses, beam stability, and linearity between the delivered monitor units (MU) and dose were previously checked with the Semiflex 0.125 cc ionisation chamber (PTW, Freiburg, Germany).

After irradiation was completed, the films were left for 24 h to allow for post-exposure density growth. Then, the films were scanned in the same orientation, as a single scan protocol, using the EPSON Perfection 750 pro flatbed scanner (EPSON, Suwa, Japan) in transmission mode. Prior to performing the scan, the scanner was warmed up and a preview scan was performed to stabilise the lamp temperature. The film pieces were scanned in the 48-bit red-green-blue mode (16 bits per colour) and saved at 72 dpi spatial resolution in the TIFF format. Film data processing was performed with the ImageJ software (National Institute of Health, Bethesda, MD, USA). The channels were split and the analysis was performed using the red channel.

### 2.5. Flow Cytometry Analysis

The DNA damage in the irradiated PNT1A cells was assessed with the Apoptosis, DNA Damage and Cell Proliferation Kit (BD Biosciences, Franklin Lakes, NJ, USA). The cells were kept at room temperature until collection. The cells were collected using the Accutase cell detachment solution (Biowest, Nuaillé, France). Cells were first washed with 1× BD Perm/Wash™ Buffer (BD Biosciences, Franklin Lakes, NJ, USA), and 5 × 10^5^ cells were fixed using the BD Cytofix/Cytoperm™ Fixation/Permeabilization Solution (BD Biosciences, Franklin Lakes, NJ, USA). The cells were fixed two hours after irradiation. Next, the cells were stained with an anti-γH2AX antibody (mouse, Alexa Fluor^®^ 647 conjugated). The stained cells were analysed using a CytoFLEX flow cytometer (Beckman Coulter Life Sciences, IN, USA) and FlowJo V10 software (FlowJo LCC, Ashland, OR, USA). For the fluorescence signal quantification, the percentage of positive cells was evaluated.

### 2.6. Clonogenic Assay

The cells were collected one hour after irradiation using the Accutase solution, resuspended in a culture medium, and counted with a MOXI Z automated cell counter (Orflo Technologies, Ketchum, ID, USA). Cells were plated onto T-25 flasks with vented caps at 500 cells for all of the cell locations (controls plus intestine, lung, thyroid, and brain), except for the prostate, for which 4000 cells were used. Cells from each biological replicate were plated in three replicates and cultured in RPMI 1640 with 10% FBS for 16 days. Next, cells were fixed in ethanol and stained using Coomassie Brilliant Blue solution (Sigma-Aldrich, St. Louis, MO, USA). To determine the colony number, the flasks were photographed using the ChemiDoc Touch Imaging System (Bio-Rad, Hercules, CA, USA) and analysed with Image Lab Software v6.0 (Bio-Rad, Hercules, CA, USA). Colonies were scored by counting clusters with ≥50 cells. Plating efficiency (PE) was determined by calculating the ratio between the number of colonies and number of plated cells. The surviving fraction was the ratio between the PE values of the irradiated and control cells.

### 2.7. RNA Isolation and Reverse Transcription

Total RNA was isolated from the PNT1A cells which were collected one hour after irradiation, plated on a 6-well plate and cultured in full medium for 24 h. After that the total RNA was isolated, using TRI Reagent (Sigma-Aldrich, St. Louis, MO, USA) and Direct-zol™ RNA MiniPrep Kit (Zymo Research, Irvine, CA, USA), according to the manufacturer’s instructions. The concentration and purity of isolated RNA were determined by gel electrophoresis and spectrophotometry, using DeNovix DS-11 (DeNovix, Wilmington, DE, USA) to measure absorbance at 260, 230, and 280 nm. The first-strand cDNA was synthesised from 1 µg of the isolated RNA using iScript™ RT-qPCR cDNA Synthesis Kit (Bio-Rad, CA, USA), following the manufacturer’s instructions. The cDNA was then diluted ten times in nuclease-free water.

### 2.8. Microarray Expression Study

The microarray study was carried out as described elsewhere [18,19,20,21]. Briefly, 100 ng of total RNA from each sample was subjected to a two-round sense cDNA amplification, followed by biotin labelling and cDNA fragmentation using the GeneChip Whole Transcript (WT) PLUS Reagent Kit (Affymetrix Inc., Santa Clara, CA, USA). Biotin-labelled fragments of cDNA (5.5 μg) were hybridised to the Affymetrix Human Gene 2.1 ST ArrayStrip (20 h at 48 °C). After hybridisation, the microarrays were washed and stained, using the Affymetrix GeneAtlas Fluidics Station (Affymetrix, Santa Clara, CA, USA). The array strips were scanned by the Imaging Station of GeneAtlas System (ThermoFisher Scientific, Waltham, MA, USA). Preliminary analysis of the scanned chips was performed, using the Affymetrix GeneAtlas Operating Software (Affymetrix, Santa Clara, CA, USA) to verify the quality of the detected signals. The Affymetrix CEL files were imported for downstream data analysis.

### 2.9. Microarray Data Analysis

The BioConductor package of R programming language was used to perform the following analyses. The Robust Multiarray Average (RMA) normalisation algorithm implemented in the “Affy” library was used to normalise, correct for background, and calculate the expression values of the examined genes [22]. Biological annotations were taken from the BioConductor “oligo” package, where the annotated data frame object was merged with the normalised dataset, leading to a complete gene data table [23]. Differential expression and statistical assessment were determined by applying the linear models for microarray data in the “limma” library [24]. The accepted selection criteria for significantly changed gene expression were based on a fold change > absolute 1.5, and an adjusted *p* value < 0.05. The results of this selection were presented as a volcano plot, showing the up- and downregulated genes. Genes fulfilling the selection criteria were considered significantly different and subjected to further analyses, described in the next section.

All raw data files were deposited in the Gene Expression Omnibus (GEO) repository at the National Center for Biotechnology Information (http://www.ncbi.nlm.nih.gov/geo/query/acc.cgi?acc=GSE205026) under GEO accession number GSE205026.

### 2.10. Gene Set Enrichment Analysis (GSEA)

GSEA was used to determine enrichment or depletion in gene expression between the study groups and the control group within a priori defined gene sets (Gene Ontology (GO) and Kyoto Encyclopaedia of Genes and Genomes (KEGG)). The method uses the Kolmogorov–Smirnov (K–S) statistical test to identify significantly enriched or depleted groups of genes [25]. Normalised fold change values from all of the genes presented on the microarray were log2 transformed and ordered. Next, a predefined gene set from the Hallmark database (from the Molecular Signatures Database) was selected [26]. Genes belonging to the selected set were ranked according to the difference in their expression level, using a signal-to-noise ratio with 1000 permutations. By walking down the ranked list of genes, the enrichment score (ES) was calculated for each selected gene set [27], and these scores were normalised by their gene set size, with false positives corrected by the false-discovery rate technique.

### 2.11. Real-Time Quantitative PCR

To validate the results of microarray analysis, we analysed the expression of selected genes that were expressed differently between variants. Gene expression was measured using the synthesised PrimePCR plates (Bio-Rad, Hercules, CA, USA) with the Fast Start Essential DNA Green Master (Roche, Basel, Switzerland) on the CFX96 Touch Real-Time PCR Detection System (Bio-Rad, Hercules, CA, USA), according to the manufacturer’s instructions. Each reaction was run in a technical duplicate. The RT-qPCR results were presented as relative mRNA expression level calculated with the 2^−ΔΔCT^ method and using β-2 microglobulin as a reference gene.

### 2.12. Statistical Analysis

Statistical analyses were performed using the GraphPad Prism software program, v.8.1.1 (GraphPad Software, Inc., La Jolla, CA, USA). For all of the datasets, the distribution normality was tested using the Shapiro–Wilk test. Data passing the normality test were analysed with the one-way ANOVA test with Tukey’s correction for multiple comparisons. Non-normal data were analysed with the Kruskal–Wallis test with Dunn’s correction. The threshold for statistical significance was set at *p* < 0.05.

## 3. Results

### 3.1. The Dose Measurement in the Locations of Peripheral Organs during Prostate VMAT SBRT Delivery

**Figure 2:**
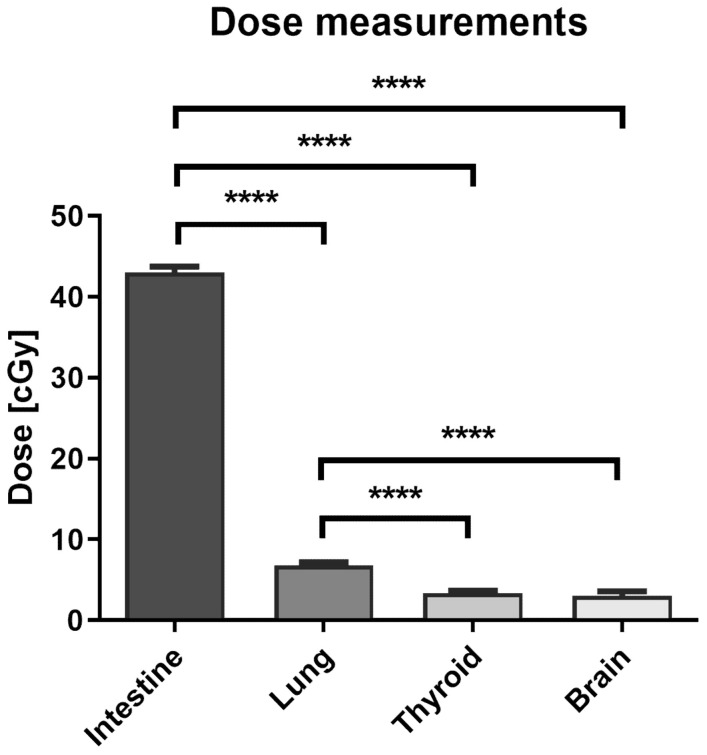
Radiation doses measured in peripheral organ locations following VMAT SBRT. Doses were measured using EBT3 Gafchromic films placed under culture flasks during irradiation. Data are shown as means ± SD for six independent experiments. ****—*p* < 0.0001, based on one-way ANOVA with Tukey’s post-hoc test.

The dose delivered to the PTV was validated using a standard dose verification used for the clinical practice, with a CAX dose of 70 Gy. Compared to the other peripheral organ locations, a significantly higher dose was registered in the intestine (15 cm from the axial plane): 0.43 Gy ± 0.001 Gy (*n* = 6). The doses decreased as the distance from the axial plane increased, as follows: lung (35 cm): 0.07 Gy ± 0.004 Gy (*n* = 6); thyroid (52 cm): 0.03 Gy ± 0.002 Gy (*n* = 6); brain (74 cm): 0.03 Gy ± 0.006 (*n* = 6) (Figure 2). The dose registered in the lung was significantly higher than the doses in more distant locations. As a percentage of the planned axis dose, doses in the peripheral organs were as follows: intestine: 0.611%; lung: 0.100%; thyroid: 0.050%; and brain: 0.048%.

### 3.2. The Effect of Prostate VMAT SBRT Delivery on DNA Damage and Cell Death in Cells Irradiated in Peripheral Organs

**Figure 3:**
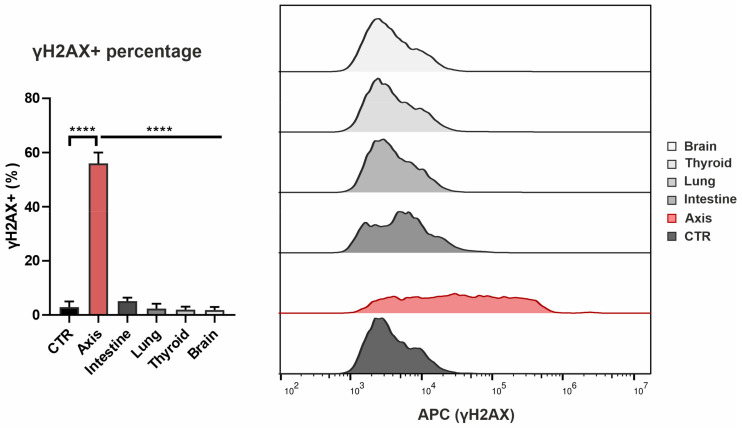
Effect of VMAT irradiation on the level of double-strand breaks in healthy human prostate cells positioned in the beam axis and peripheral organs. The level of DNA double-strand breaks was measured with flow cytometry using the γH2AX marker. The percentage of γH2AX-positive PNT1A cells was measured for irradiated cells in each peripheral organ location. Data are shown as means ± SD of three independent experiments. ****—*p* < 0.0001, based on one-way ANOVA with Tukey’s post-hoc test.

The DNA damage in the prostate cells irradiated in the peripheral organ locations was evaluated by cytometric measurement of phosphorylated histone H2AX (γH2AX) levels in the cells. Histone H2AX is phosphorylated directly following the double strand-break (DSB) of DNA and is often used as a marker of the genotoxic effect of ionising radiation [28]. As expected, we observed a significant increase in the γH2AX-positive population for the cells located in the axis (control: 2.89% ± 2.10% vs. axis: 55.97% ± 4.04%; *n* = 3), but not in the cells located out of the field (Figure 3). Although an increase in the γH2AX-positive population was observed in the intestine (control: 2.89% ± 2.10% vs. intestine: 5.09% ± 1.32%; *n* = 3), the difference was not statistically significant.

**Figure 4:**
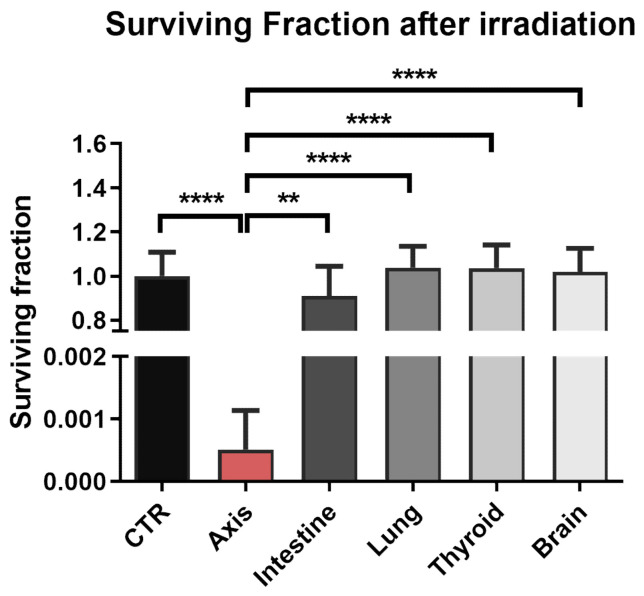
Effect of VMAT SBRT irradiation on survival of healthy prostate cells positioned in the prostate and peripheral organs. Survival was measured using clonogenic assay. The surviving fraction of these cells compared to sham-irradiated cells was measured for each location. Data are shown as means ± SD of three independent experiments, normalised to the mean of the sham-irradiated cells (CTR). **—*p* < 0.01, ****—*p* < 0.0001, based on one-way ANOVA with Tukey’s post-hoc test.

Next, we measured the surviving fraction (SF) of prostate cells irradiated in the axis and peripheral organs using a clonogenic assay. Similar to the assessment of DNA damage, clonogenic survival was significantly decreased only in the cells in the beam axis (SF = 0.0005 ± 0.0006; *n* = 18) (Figure 4). Cell survival decreased in the intestine vs. controls (control: SF = 1.0 ± 0.11 vs. intestine: SF = 0.91 ± 0.14; *n* = 18), although the difference was not statistically significant.

### 3.3. The Effect of Prostate VMAT SBRT Delivery on the Transcriptomic Profile of Cells Irradiated in Peripheral Organs

**Figure 5:**
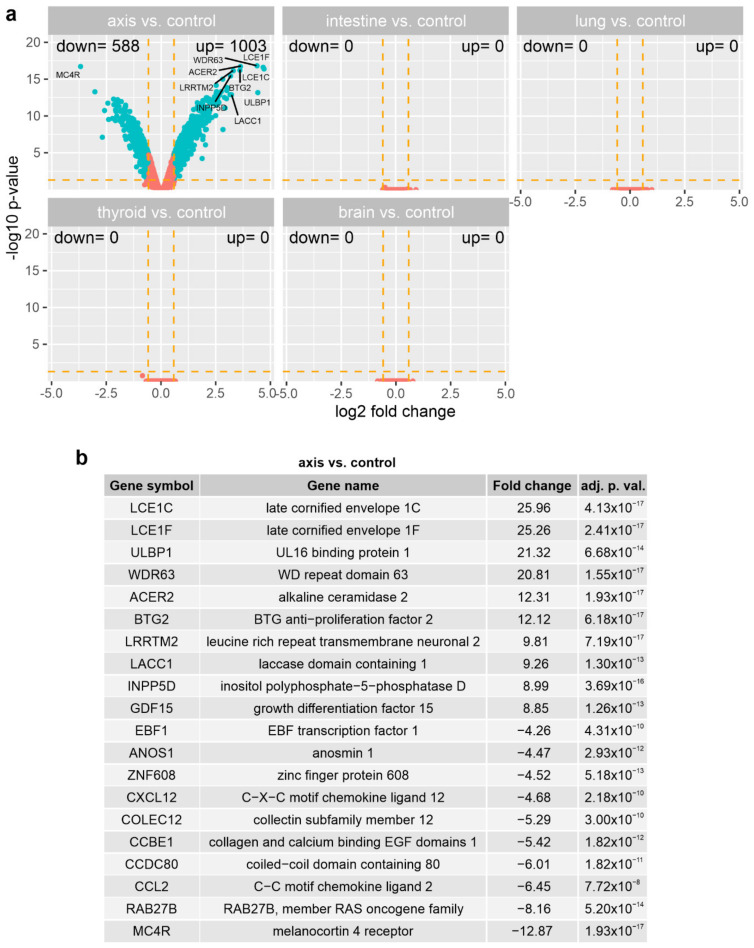
The transcriptomic profiles of prostate cells irradiated in the beam axis and peripheral organs. (**a**) Volcano plots depicting expression profiles of the experimental groups. Each dot represents the mean expression (three biological replicates) of an individual gene obtained from a normalised microarray dataset. The dotted orange lines (cut-off values) were established according to the following parameters: |fold change| = 1.5 and *p* value = 0.05. Genes above the cut-off lines were considered as differentially expressed genes and are shown as blue dots. The total numbers of up- and down-regulated genes are given in the top right and top left corners, respectively. The ten most differentially expressed genes are marked on the plot; (**b**) 20 genes with the highest (10 genes) and the lowest (10 genes) fold change from the list of genes differentially expressed between the cells irradiated in axis and controls.

We used the GeneChip™ Human Gene 2.1 ST Array Strip (Affymetrix Inc., Santa Clara, CA, USA) to evaluate the differences in expression of over 30,000 human transcripts in the PNT1A cells irradiated with VMAT SBRT in the beam axis and peripheral organs. The transcriptome study was performed on the cells collected 24 h after irradiation, with sham-irradiated cells used as controls. The general profile of the transcriptome changes is shown in Figure 5. Each dot represents the mean gene expression (three biological repeats). We used the following cut-off criteria to indicate significantly changed expression: |fold change| > 1.5, and *p* value < 0.05. The only significant difference in gene expression was observed between the cells irradiated in the axis vs. controls. The ten most highly-expressed genes and the ten most decreased genes are shown in Figure 5b. The fold change values of the top ten upregulated genes in the cells irradiated in the beam axis vs. the control cells ranged from 8.85 to 25.96. The fold change values of the top ten downregulated genes in the axis-located cells vs. controls ranged from −4.26 to −12.87. The top ten upregulated genes in the axis cells compared to controls were: late cornified envelope 1C (LCE1C); late cornified envelope 1F (LCE1F); UL16 binding protein 1 (ULBP1); WD repeat domain 63 (WDR63); alkaline ceramidase 2 (ACER2); BTG anti−proliferation factor 2 (BTG2); leucine-rich repeat transmembrane neuronal 2 (LRRTM2); laccase domain containing 1 (LACC1); inositol polyphosphate−5−phosphatase D growth (INPP5D); and differentiation factor 15 (GDF15). The top ten downregulated genes in the axis cells compared to controls were: EBF transcription factor 1 (EBF1); anosmin 1 (ANOS1); zinc finger protein 608 (ZNF608); C−X−C motif chemokine ligand 12 (CXCL12); collectin subfamily member 12 (COLEC1); collagen and calcium binding EGF domains 1 (CCBE1); coiled−coil domain containing 80 (CCDC80); C−C motif chemokine ligand 2 (CCL2); RAB27B; member RAS oncogene family (RAB27B); and melanocortin 4 receptor (MC4R). Overall, the analysis revealed 1003 upregulated and 588 downregulated genes in the cells irradiated in the axis compared to the sham-irradiated controls. No significant differences were observed in the expression of transcripts in the cells irradiated in the other locations (intestine, lung, thyroid, and brain) compared to controls.

### 3.4. The Effect of Prostate VMAT SBRT Delivery on Enrichment of Biological Processes in Cells Irradiated in Peripheral Organs

**Figure 6:**
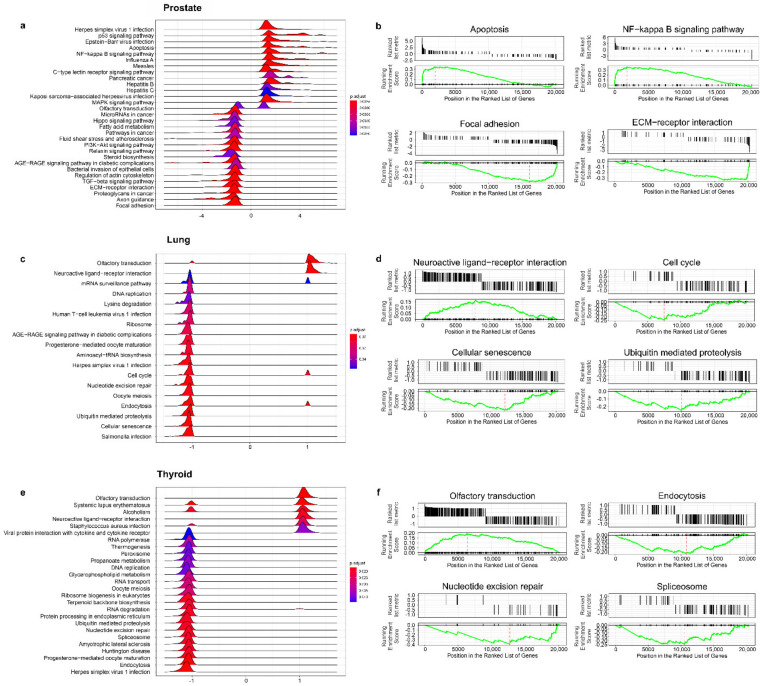
Results of gene set enrichment analysis (GSEA) based on gene expression measured in cells irradiated in prostate and peripheral organ locations. (**a**,**c**,**e**) show ridgeline plots with expression distribution for pathways differentially enriched between samples. The plot displays only the distribution of core enriched genes; (**b**,**d**,**f**) show the GSEA enrichment plots of selected gene sets. The peak of the green plot marks the enrichment score (ES) of a process. This analysis was based on the expression of all of the measured genes, both differentially expressed and non-differentially expressed.

To analyse the differences in gene expression within predefined gene sets, the data were subjected to GSEA. For this analysis, we included both of the genes above, and below the cut-off of |fold change| = 1.5 and *p* = 0.05. First, the fold values of all of the genes were log2 transformed and ranked, based on the log2 fold change. These values were used to calculate the Enrichment Score (ES-a permutation test run 1000 times) within predefined gene sets from the KEGG database. The core-enriched genes for the GSEA-enriched pathways in the cells irradiated in the prostate are shown on a ridgeline plot (Figure 6a). Some of the top enriched terms related to the DNA damage response to ionising radiation, and included the overrepresented “Apoptosis” and “NF-kappa B signalling pathway”, and depleted “Focal adhesion” and “ECM-receptor interaction” (Figure 6b).

The GSEA analysis revealed no differently enriched pathways in the cells in the intestine and brain compared to the controls. Interestingly, this analytical approach revealed differences in the enrichment of gene sets between the irradiated cells in the lung and thyroid. In the cells in the lung, the analysis showed upregulation of “Neuroactive ligand-receptor interaction” and downregulation of (among others) “cell cycle”, “cellular senescence”, and “ubiquitin mediated proteolysis” gene sets (Figure 6c,d). For the cells in the thyroid, the analysis revealed upregulation of “olfactory transduction” and downregulation of “endocytosis”, “nucleotide excision repair”, and “spliceosome” gene sets (Figure 6e,f).

**Figure 7:**
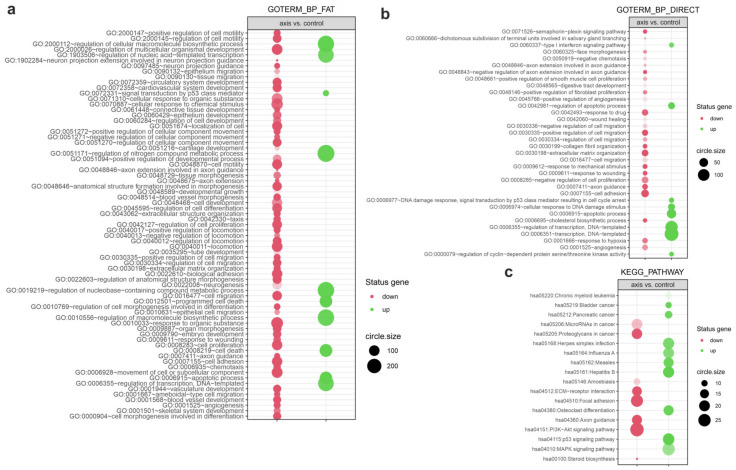
The processes differently regulated following irradiation of cells in the prostate. The bubble plot shows differently expressed gene sets in various databases—(**a**) DAVID GO BP FAT database; (**b**) DAVID GO BP DIRECT database; (**c**) KEGG PATHWAY database—that were expressed differently between PNT1A cells irradiated in the axis vs. controls. The cut-off criteria were: |fold change| > 1.5 and (**a**) *p* value < 5 × 10^−6^; (**b**,**c**) *p* value < 0.05. The red and green bubbles represent downregulated and upregulated gene sets, respectively. The bubble size represents the number of genes regulating the gene set. The transparency of the bubble represents the *p* value (greater transparency denotes *p* values closer to the cut-off value). This analysis was based only on the differentially expressed genes.

Given that the GSEA analysis considers only enrichment in sets of genes, to determine the biological processes that were differentially regulated after irradiation we assessed enrichment in the relevant ontological groups from Gene Ontology—Biological Process (GO BP) Direct, GO BP Fat, and Kyoto Encyclopaedia Genes and Genomes (KEGG) PATHWAY databases. This analytical method considers not only gene expression within a set, but also the interactions and dependencies between genes. For this analysis, only the differentially expressed genes were considered (fold change > 1.5 and *p* value < 0.05). Differentially expressed genes were assigned to the GO BP Fat database. The analysis revealed an altered regulation of several processes in the cells irradiated in the prostate compared to the controls (Figure 7a); specifically, upregulation of the following: “signal transduction by p53 class mediator (GO:0072331)”, “programmed cell death (GO:0012501)”, “cellular death (GO:0008219)”, and “apoptotic process (GO:0006915)”; and downregulation of “positive regulation of cell motility (GO:2000147)”, “positive regulation of cell migration (GO:0030335)”, “biological adhesion (GO:0022610)”, and “cell proliferation (GO:0008283)”.

Next, we assigned the differently expressed genes to the GO BP Direct database, which revealed changes in the regulation of multiple processes in the cells in the prostate compared to control cells (Figure 7b), as follows: upregulation of “regulation of apoptotic process (GO:0042981)”; “cellular response to DNA damage stimulus (GO:0006974)”; “apoptotic process (GO:0006915)”; and “transcription, DNA-templated (GO:0006351)”; and downregulation of “cell adhesion (GO:0007155)”; “negative regulation of cell proliferation (GO: 0008285)”; and “regulation of cell migration (GO:0030334)”.

The differentially expressed genes were also assigned to the KEGG PATHWAY database, which determined several biological processes that were differentially regulated in the prostate cells in the axis vs. controls (Figure 7c). We observed changes in the regulation of multiple processes: upregulation of “p53 signalling pathway (hsa04115)” and “MAPK (mitogen-activated protein kinase) signalling pathway (hsa04010)”; and downregulation of “MicroRNAs in cancer (hsa05206)”; “ECM-receptor interaction (hsa04512)”; “Focal adhesion (hsa04510)”; and “PI3K-Akt signalling pathway (hsa04151)”.

This analysis revealed no significant differences in the regulation of relevant processes for the cells irradiated in peripheral organs compared to the sham-irradiated controls.

**Figure 8:**
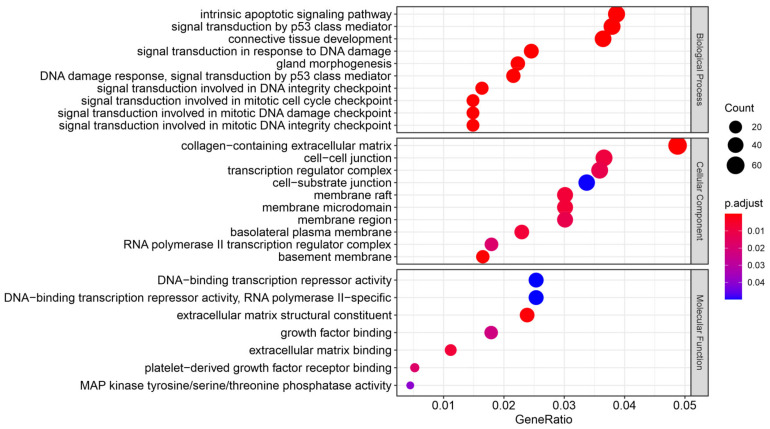
Dot plot of enriched GO terms of differentially expressed genes in cells irradiated in the prostate vs. the sham-irradiated cells. The enriched GO terms belong to three domains: biological process, cellular component, and molecular function. The gene ratio denotes a relative abundance of genes in a GO term. The dot size represents the number of enriched genes in a GO term. The colour represents the adjusted *p* value. This analysis was based only on the differentially expressed genes.

GO pathway analysis of the enriched functional pathways in the biological process domain revealed overrepresentation of gene products involved with (among others) “intrinsic apoptotic signalling pathway” and “signal transduction by p53 class mediator” in the cells irradiated in the prostate. Several processes related to the response to ionising radiation were also enriched, including: “signal transduction in response to DNA damage”, “DNA damage response, signal transduction by p53 class mediator”, “signal transduction involved in DNA integrity checkpoint”, “signal transduction involved in mitotic cell cycle checkpoint”, “signal transduction involved in mitotic DNA damage checkpoint”, and “signal transduction involved in mitotic DNA integrity checkpoint” (Figure 8). The analysis within the cellular component domain showed that the products of genes differentially expressed in the cells irradiated in the prostate were active within the following: “collagen-containing extracellular matrix”; “cell-cell junction”; and “transcription regulator complex”. In the cells irradiated in the prostate, we observed enrichment in the molecular function domain of the following: “DNA-binding transcription repressor activity”; “DNA-binding transcription repressor activity, RNA polymerase II-specific”; and “extracellular matrix structural constituent”.

**Figure 9:**
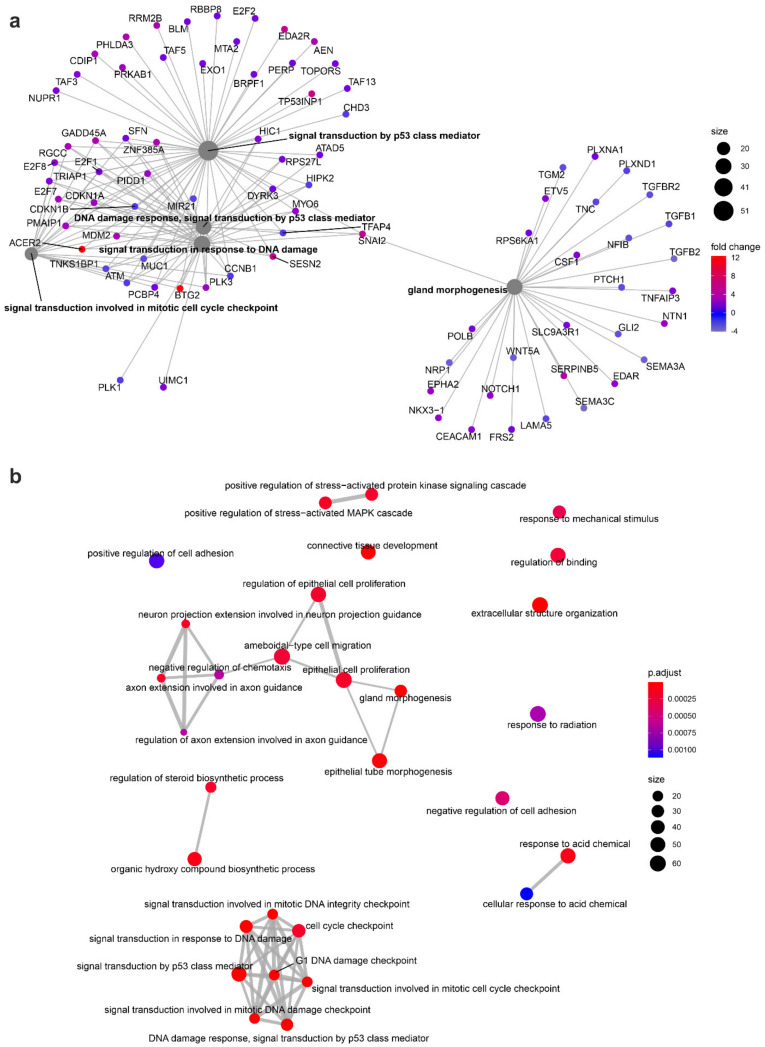
Visualisation of the relationships between biological processes and genes differentially regulated in cells irradiated in the prostate vs. the sham-irradiated cells. (**a**) Network plot showing linkages of genes and biological processes differentially expressed between irradiated and sham-irradiated cells in the prostate. The colour of the bubble represents a fold expression change between prostate and control. The size of the process bubble represents a number of differentially expressed genes; (**b**) Enrichment map showing relationships between processes enriched in cells irradiated in prostate compared to sham-irradiated. The size of the process bubble represents a number of differentially expressed genes. Lines connecting enriched processes are weighted by the radio of overlapping gene sets. This analysis was based only on the differentially expressed genes.

To visualise the genes associated with the biological process domain of the GO terms in the cells irradiated in the prostate, we created a figure (Figure 9a) to visualise the linkages of the genes and enriched processes as a network plot of enriched terms. For this analysis, only the differentially expressed genes were considered (fold change > 1.5 and *p* value < 0.05). The plot shows connections between enriched genes that were related to the following processes: “signal transduction by p53 class mediator”; “DNA damage response, signal transduction by p53 class mediator”; “signal transduction in response to DNA damage”; and “signal transduction involved in mitotic cell cycle checkpoint”. The “gland morphogenesis” process was also enriched in the irradiated cells, but only weakly correlated with the other four processes.

To visualise the relationships between biological processes, we created an enrichment map with the enriched terms (Figure 9b). Figure 9 shows a functional module of terms related to epithelial cell proliferation and regulation of neuron growth (middle of map), and a second functional module of terms related to DNA damage response (terms such as: “signal transduction by p53 class mediator”; “cell cycle checkpoint”; “DNA damage response, signal transduction by p53 class mediator”).

**Figure 10:**
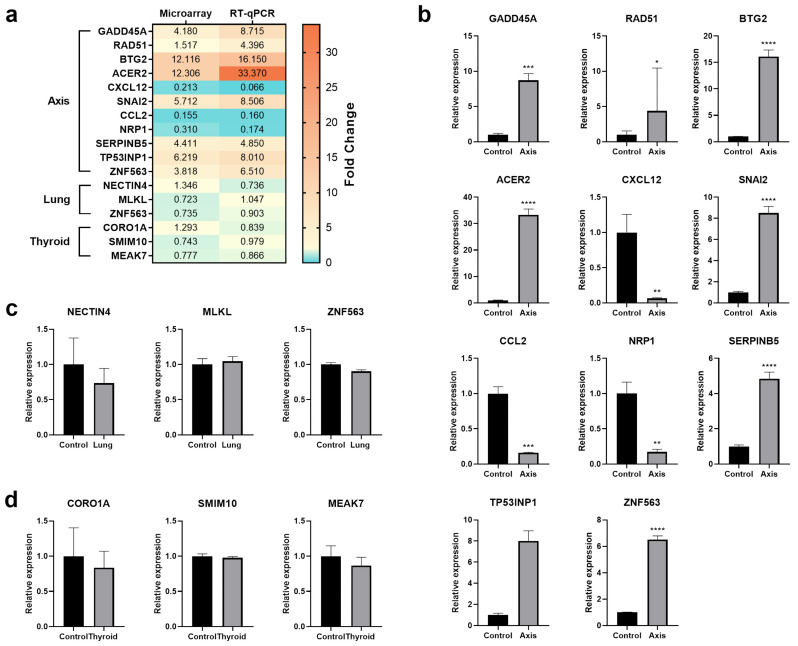
Real-time qPCR validation of microarray data. (**a**) Heat map showing fold change expression of chosen genes, measured with microarray and RT-qPCR; (**b**) The expression of selected genes in cells irradiated in the prostate (axis) measured using the RT-qPCR technique; (**c**) The expression of selected genes in cells irradiated in lung measured using the RT-qPCR technique; (**d**) The expression of selected genes in cells irradiated in thyroid measured using the RT-qPCR technique. The graphs represent means ± SD from three biological replicates. *—*p* < 0.05, **—*p* < 0.01, ***—*p* < 0.001, ****—*p* < 0.0001, based on Student’s *t*-test.

The results of the microarray analyses were verified by RT-qPCR (Figure 10). For the verifications, we chose genes that showed the highest and lowest fold changes (axis—BTG2, ACER2, CXCL12, CCL2; lung—NECTIN4, MLKL, ZNF563, thyroid—CORO1A, SMIM10, MEAK7) and genes that were included in several processes differentially regulated in the investigated cells (axis—GADD45A, RAD51, SNAI2, NRP1, SERPINB5, TP53INP1, ZNF563).

## 4. Discussion

We sought to measure the biological response of the cells in the peripheral organs located outside of the radiation field during the delivery of gantry-based VMAT SBRT prostate radiotherapy. In our study we measured the response in organs lying further from the target, while other studies focused on toxicity in the bladder and rectum. We investigated in vitro the biological effects induced in the peripheral organs in a phantom-based model. Briefly, for this experiment, the healthy prostate cells (PNT1A) were irradiated in a quasi-humanoid water phantom in locations corresponding to the prostate, intestine, right lung, thyroid, and brain. The prescribed dose per fraction was 10 Gy (the highest fraction dose used for prostate SBRT) repeated without intermission seven times to a total dose of 70 Gy (scaling-up method). The use of a dose scaling approach allowed us to observe higher deposition in intestine and lung compared to the more distant locations. We were able to show a strong, multiparametric radiobiological response of the cells in the prostate, which included increase in DNA damage, decrease in cell survival, and enrichment of several biological processes related to DNA damage response. We observed no significant biological effect in the out-of-field peripheral organs. In the intestine we found a minor increase in DNA damage and cell death, however no statistical significance was proved. In the lung and thyroid, a depletion of a few gene groups was observed, however the groups were not clearly linked with response to ionising radiation.

The EBT Gafchromic films used in this study have a slightly different effective atomic number than human tissue. As a result, doses read with this method can differ from the delivered doses. The EBT3 films were shown to over-respond to irradiation with low energy photons by up to 10% [29]. Scattered photons out-of-field have a significantly lower energy than in-field energy, and it is reasonable to assume that some small variability in dose readings might be present in this study; however, the exact values are not clear. The dose readings from the EBT films placed under the culture flasks showed a mean dose of 0.43 Gy (0.611% of the axis dose) in the intestines and of 0.07 Gy (0.1% of the axis dose) in the lung. The doses in both locations were significantly higher than doses registered in more distant locations, such as the thyroid (0.03 Gy; 0.05% of the axis dose), and brain (0.03 Gy; 0.048% of the axis dose).

In a previous study, our group investigated out-of-field doses in a water phantom from a static 10 cm × 10 cm field delivered with a 6 MV photon beam [30]. In that study, the doses registered with radiochromic films for the static flattening filter-free (FFF) field were lower than the doses observed in the present study: the dose at 15 cm from the axis was 1.1% of the total dose (vs. only 0.611% in the present study) and the dose at 35 cm from the axis was 0.03% of the total dose (vs. 0.44%). This difference suggests that conformal radiotherapy results in lower doses close to the field but higher doses further away, when compared to static field delivery techniques. These differences in registered doses might also be attributable to the use of a flattening filter. Although the FFF technique is the most common approach in prostate radiotherapy, because of the lower peripheral doses, in the current study we applied a flattened beam to achieve a more uniform dose profile with higher doses further away from the treatment field [31]. Kry et al. used a Monte Carlo simulation to estimate the out-of-field doses from 6 MV IMRT delivered with a Varian 2100 accelerator (using different treatment modalities) [32]. Those authors observed that the FFF techniques resulted in lower doses in the areas close (<3 cm) and far (>15 cm) from the treatment field; however, in the area between these distances, the doses were higher than observed when radiation was performed with the flattening filter. Treutwein et al. used an Alderson phantom to measure doses in patients with PCa treated with 6 MV IMRT and VMAT with and without a flattening filter. The measurements were performed after a single fraction and based on a treatment plan of 33 fractions (minimum dose of 71 Gy to the CTV) [33]. Overall, the VMAT treatment doses were lower than the IMRT doses (both with and without the flattening filter). The percentage CTV doses for VMAT with the flattening filter were as follows: 0.16% in the colon region; 0.05% in the oesophagus region; and 0.06% in the thyroid region; the corresponding percentages for FFF VMAT were 0.12%; 0.03%; and 0.03%.

Many dosimetric studies have shown that VMAT SBRT provides satisfactory target coverage and sparing of OARs. To our knowledge, however, there are no studies that have investigated the biological effects of this treatment modality on organs located more distant from the prostate. Our analysis of the DNA DSBs in the different phantom locations showed that VMAT induced significant damage in the prostate but not in the peripheral organs. Cells in the intestine region showed a small increase in the fraction of the γH2AX-positive cells compared to the sham-irradiated cells; a non-significant increase was also observed in the cells in the other peripheral organs.

The DSB level correlated with cell survival, with a significant reduction in the SF in the cells irradiated in the beam axis, and a small (but non-significant) decrease in the cells in the intestine. This finding suggests that VMAT SBRT for the prostate probably offers a sufficient sparing of the peripheral organs located at distances from the beam axis that are greater than or equal to the distance from the lungs; however, scatter radiation could damage the intestines. While there is no clear consensus between studies in terms of the optimal dose constraints to limit the radiotoxic effects in the large intestine, the constraints proposed by Jadon et al. in their systematic review involve doses that are significantly higher than those observed in our study [34]. The crypt stem cells present in the intestine are radiosensitive, but studies show that these cells can regenerate after radiation exposure of up to several Gy [35]. In our study, a single escalated dose of VMAT SBRT resulted in a notable dose deposition in the intestines; nevertheless, the measured dose would probably be too low to induce clinically significant acute or late effects in this organ. The minor biological effect observed in the cells in the intestine (low level of DSBs, a slight decrease in cell survival) confirms that the radiotoxic effects of this modality to the intestine or more distant organs are minuscule.

Studies have shown that even very low doses of radiation (<50 mGy) can induce DNA damage in the cells [36], although we did not observe any significant increase in DNA damage or cell death in the cells in the lung where the registered dose was 69.7 mGy, nor in the more distant locations which were exposed to lower doses. The cellular response we observed could be attributed to several different factors. First, given that the total radiation dose of 70 Gy was delivered over approximately 50 min, the effective dose rate in the peripheral organs was low, which would explain the minimal cell damage (higher dose rates are associated with greater DNA damage) [36]. Importantly, this effect can differ significantly depending on the origin of the cell lines [37]. A previous study by our group demonstrated that the out-of-field energy from a 6 MV photon beam drops rapidly between the edge to 10 cm beyond the field edge; however, the energy values are similar in the region from 10 to 35 cm outside the field edge [8].

Interestingly, in a study based on Monte Carlo modelling, Kirkby et al. suggested that a 6 MV photon beam might have a greater relative radiobiological effectiveness outside of the field, due to the higher relative component of low energy radiation [7]. Syme et al. confirmed that the scattered radiation from a 6 MV beam induces more DNA damage at the same delivered dose than open beam radiation [38]. Liu and Verhaegen showed that the beam quality factor of a 6 MV photon beam could vary by up to 20%, due to energy spectrum changes [39], which is especially relevant to VMAT techniques as the field size and shape are modulated throughout delivery, thus impacting the energy and dose delivered out-of-field. By contrast, in a study that measured both in- and out-of-field doses from a 6 MV VMAT beam in a water phantom, Shields et al. found that the cells irradiated 1 cm out-of-field exhibited fewer DSBs compared to the cells that received the same dose in-field [10]. In a previous in vitro study conducted by our group (20 MV photon beam), we found that the biological response of the cells irradiated in and out-of-field differed significantly, with a stronger response observed in the cells located within the field [40]. In the present study, the doses registered in the peripheral organ locations were too low to induce acute or late toxicity; nevertheless, research shows that even radiation doses that are too low to induce severe cellular damage can still increase the risk of secondary cancers, especially in radiosensitive organs, such as the thyroid [41,42].

We found higher radiation doses in the intestines and lungs compared to more distant organs. Given the slight increase in damage to the cells in the intestines, we decided to determine whether gene expression was altered in these irradiated cells. Based on the existing research on low-dose radiation-induced changes in mRNA expression, we expected that using microarray assay would allow us to detect significant changes in expression induced by doses as low as 10–50 mGy, if those were present in our samples [43,44]. Our analysis of expression changes showed that VMAT to the beam axis induced processes related to cell death, apoptosis, p53 signal transduction, response to DNA damage induced by radiation, and depleted processes such as cell proliferation, adhesion, and migration.

The enriched NF-kappa B signalling pathway was also associated with healthy tissue toxicity following radiotherapy [45]. Transcription of NF-kappa B can be induced by damage-associated molecular patterns (DAMP), which are released from the cells following necrotic death induced by high dose radiation [46]. Upregulation of NF-kappa B can also lead to the generation of nitric oxide, which in turn can cause inflammation and inhibition of DNA repair enzymes, thus further contributing to the radiotoxic effect [45]. No differences in gene expression or enrichment of biological processes were observed in the irradiated cells in the intestine and brain compared to the sham-irradiated cells. In the intestine, this lack of enrichment of expression is coupled with a small increase in DNA damage and cell killing. Research shows that changes in the expression of genes related to the DNA damage response occur early following the exposition to radiation, and are often resolved by 24 h [47]. Persistence of radiation damage longer than 24 h can correlate with the ability of the cells to recover [48]. Coincidence of low-level DNA damage at 1 h with no changes in expression enrichment at 24 h can be a result of sufficient repair of radiation damage. 

Although the gene expression analysis revealed no differentially expressed genes in the cells irradiated in the lung and thyroid compared to controls, the GSEA analysis allowed us to identify several enriched gene groups in those two locations. In the lung, these processes included downregulation of “DNA replication”, “cell cycle”, “cellular senescence”, and “ubiquitin mediated proteolysis”. In the thyroid, the depleted gene sets included “DNA replication”, “endocytosis”, “nucleotide excision repair”, and “spliceosome”. The downregulated processes in these two groups suggest a decrease in cellular metabolism. Although changes in several gene groups were observed in the cells in these two locations, the majority of enriched groups were not clearly linked to the response to ionising radiation. We speculate that the small change in enrichment of specific gene groups in thyroid, and not the brain, which received a very similar dose, might be related to the quality of radiation in each of those locations. As mentioned above, the out-of-field radiation from a 6 MV beam can have different biological effectiveness depending on the changing energy outside the field [7]. Our previous research also showed a component of neutron radiation outside of the treatment field for the 6 MV beam [30]. With complex methods using multiple entry points |MKM|, the scatter of the beam changes with the position of the beam, which can have an impact on the quality of the radiation in different locations out-of-field. This causes the biological effectiveness of such radiation to vary, which can be reflected in the cell response.

The available research on the biological effects of prostate SBRT on healthy tissue is largely limited to the clinical and biological effects observed in organs located close to the target, and few studies have investigated the biological effects at distances greater than 20 cm from the field. Studies showed that VMAT SBRT was associated with a low incidence of gastrointestinal (GI) toxicity, but a relatively high risk of genitourinary (GU) toxicity [49,50]. The effects of radiation on more distant organs are often more difficult to evaluate since the radiation doses in those organs are too low to induce organ toxicity. However, since radiation can induce DNA damage at even low doses, potentially leading to stable genetic alterations, and eventually carcinogenesis, the development of secondary cancers can be considered an adequate measure of risk to peripheral organs following radiotherapy. Several studies reported a significant increase in secondary cancer rates in patients treated for prostate cancer with radiotherapy. Mohamad et al. recently evaluated secondary cancer risk in patients who underwent radiotherapy or surgery for localised prostate cancer between 1994 and 2012 [51], showing that photon radiotherapy had a higher overall risk than surgery; however, when specific locations were taken into consideration, photon radiotherapy was only associated with a significant increase in bladder cancer. Other studies have confirmed the increased incidence of bladder cancer after radiotherapy, with some studies demonstrating that prostate EBRT is associated with a higher incidence of lung cancer than radical prostatectomy [52,53]. Murray et al. suggested that the risk of secondary primary cancers following prostate radiotherapy depends on the use of a flattening filter [11]. In that study, the authors irradiated a RANDO phantom (6 MV VMAT) with or without a flattening filter to determine the risks to different organs based on the received dose. Their findings showed that the filter was associated with a higher risk of a secondary cancer in organs located outside the field, with the greatest increase in organs located more distant from the field than the distance to the thyroid; nevertheless, the calculated excess absolute risks of secondary malignancy were still very low (maximum of 0.62 per 10,000 persons per year). Although we found some changes in the enrichment of gene groups, such as cell cycle and nucleotide excision repair in the lung and thyroid, we did not find enrichment in any specific biological pathways or processes. The observed change in the enrichment of gene groups might be due to radiation hypersensitivity at a low dose range (doses < 0.5 Gy).

Although the in vitro model we chose for this study was designed to correspond to the biological effects observed in different locations in the human body, the alternative approach would be to use healthy cell lines specific to each peripheral organ. We used a single line of the normal prostate cells to measure the biological response of the cells. If we had used more than one cell line, this could have influenced the comparability of results from different locations, based on the individual radiosensitivity of each cell line. Our approach allowed us to directly compare the biological response of the cells irradiated in these different locations, which was the aim of the study. Additionally, the design we used was meant for the measurement of radiation effects on the cells (whether in- or out-of-field), and not for assessing the non-targeted effects (NTE). NTE, such as secretion of biological factors by the irradiated cells and direct communication through gap junctions propagate the effects of radiation, are especially relevant for low-dose exposures [54]. In our experiments, the cells were able to secrete factors to their surroundings for up to one hour after irradiation, after which the cells were washed and collected. This prohibits the propagation of NTE. In order to measure the NTE, a setup with separation of the irradiated cells and non-irradiated cells should be constructed [55], however, the investigation of NTEs specifically was outside the scope of this study.

For this study, the approach of dose-scaling to the total dose of 70 Gy was used, instead of a single SBRT fraction dose of 10 Gy. The doses out-of-field resulting from irradiation with a single fraction dose (up to 10 Gy) are very low, which makes them hard to quantify with high confidence. By contrast, the “scaling up” scheme used in our experiment delivered doses to the peripheral organs that were sufficiently high for detection with EBT films, and allowed the comparison of effects induced in different locations. Since in our experiment radiation was delivered by repeating the delivery of one fraction dose, the biological effectiveness of radiation out-of-field was the same as it would be with the use of a single dose. It should also be stressed that the effects we observed were that of the higher dose. Although the differences in biological response are generally greater when different doses up to several Gy are considered, we expect the dose used in this study to induce different effects than a single fraction dose of 10 Gy [56].

## 5. Conclusions

This study provides comprehensive multiparametric data on cellular processes that take place during the delivery of prostate VMAT SBRT in the locations of peripheral organs. The irradiated cells in the prostate presented a strong radiobiological response, including a significant increase in the DSBs, cell death, and enriched involvement of signalling pathways associated with response to radiation.

The radiation dose in the intestine was significantly higher than in sites located further from the axial plane, and the cells irradiated in the intestine also exhibited higher levels of DSBs and cell death compared to the sham-irradiated cells, although these differences were not significant.

While no changes in DSB level and cell survival were observed in the lung and thyroid, several gene groups were enriched in these cells. Most of the enriched groups showed no clear association with the response to ionising radiation, and no enrichment of specific biological processes was observed, suggesting a minor biological effect in these locations.

Further research on the topic of out-of-field radiation effects of prostate VMAT SBRT is needed to elucidate whether the impact of radiation on peripheral organs should be considered as clinically significant. For that goal, additional methods with high sensitivity in detecting the biological effects of radiation should be considered. Research shows that methods, such as the detection of chromosomal aberrations and comet assay, could probably detect radiotoxic effects of doses as low as 0.01 Gy [57,58]. Additionally, assays implementing the measurement of stable genetic changes could indicate whether the out-of-field exposures could carry the risk of secondary cancer development in those organs.

## Data Availability

The microarray data presented in this study are openly available in the Gene Expression Omnibus (GEO) repository at the National Center for Biotechnology Information at http://www.ncbi.nlm.nih.gov/geo/query/acc.cgi?acc=GSE205026, reference number GSE205026.
